# Hybrid endovascular treatment for complicated aortic dissection concomitant with true lumen obliteration: a case report

**DOI:** 10.1093/ehjcr/ytae068

**Published:** 2024-01-30

**Authors:** Kuniyasu Ikeoka, Hiroyuki Nishi, Yasunori Ueda, Haruya Yamane, Yasushi Matsumura

**Affiliations:** Cardiovascular Division, NHO Osaka National Hospital, 2-1-14 Hoenzaka, Chuo-ku, Osaka, Osaka 540-0006, Japan; Cardiovascular Surgical Division, NHO Osaka National Hospital, 2-1-14 Hoenzaka, Chuo-ku, Osaka, Osaka 540-0006, Japan; Cardiovascular Division, NHO Osaka National Hospital, 2-1-14 Hoenzaka, Chuo-ku, Osaka, Osaka 540-0006, Japan; Cardiovascular Division, NHO Osaka National Hospital, 2-1-14 Hoenzaka, Chuo-ku, Osaka, Osaka 540-0006, Japan; Cardiovascular Division, NHO Osaka National Hospital, 2-1-14 Hoenzaka, Chuo-ku, Osaka, Osaka 540-0006, Japan

**Keywords:** Aortic aneurysm, Endovascular treatment, Thoracic endovascular aortic repair, Covered endovascular reconstruction of aortic bifurcation, Double D-shape moulding technique, Case report

## Abstract

**Background:**

Thoracic endovascular aortic repair (TEVAR) has been widely introduced. However, unestablished transfemoral approach due to true lumen obliteration disables endovascular option.

**Case summary:**

A 74-year-old male with a history of 15-year-ago type B aortic dissection presented with chronic bilateral lower extremity claudication. CT angiography revealed that a large entry tear was located at distal to the left subclavian artery. The thoracic aneurysmal degeneration progressed and eventually required repair. True lumen of infrarenal aorta to bilateral common iliac arteries was totally collapsed by false lumen, and the re-entry tear was open at external iliac artery. Initially, we performed recanalization to the collapsed true lumen. Bidirectional approach was taken from right brachial and bifemoral arteries. The covered endovascular reconstruction of aortic bifurcation (CERAB) technique and double D-shape moulding technique (DDMT) was performed to create covered stent configuration. As secondary treatment, 1-debranching TEVAR with axillary artery bypass was successfully performed by utilizing femoral approach.

**Discussion:**

This case demonstrated feasibility of two-stage endovascular therapy for thoracic aneurysmal degeneration concomitant with true lumen obliteration. This combined technique of CERAB and DDMT was absolutely effective to minimize type Ⅲ endoleak in infrarenal segment. Hybrid endovascular treatment offered minimally invasive therapy to the patient.

**Learning points**

- The manifestation of thoracic aneurysmal degeneration concomitant with abdominal aortic true lumen obliteration is rare.
- The multidisciplinary endovascular techniques enable minimally invasive treatment.
- The covered endovascular reconstruction of aortic bifurcation technique combined with double D-shape moulding technique is effective to minimize type Ⅲ endoleak.

## Introduction

In type B aortic dissection, thoracic aorta shows a high incidence of aneurysmal degeneration during follow-up. Especially, the presence of blood flow in the false lumen is a risk factor for aortic enlargement.^1^ Thoracic endovascular aortic repair (TEVAR) has become a first-line treatment of descending thoracic aortic aneurysmal degeneration.^2^ Although transfemoral approach is commonly adopted in TEVAR, access route diseases disable catheter approach.

Lower limb malperfusion (LLM) syndrome occurs in up to 5–12% in type B aortic dissection.^3^ The LLM is one of the major concomitant problems in type B aortic dissection, and typically presents as acute limb ischaemia.^4^ In a minority of cases, chronic limb threatening ischaemia occurs by anatomically complicated limb malperfusion. In this report, we describe the novel endovascular technique of a complicated chronic type B aortic dissection concomitant with true lumen occlusion of infrarenal aorta to bilateral common iliac arteries in order to restore bilateral lower limb perfusion and establish TEVAR approach.

## Summary figure

**Figure:**
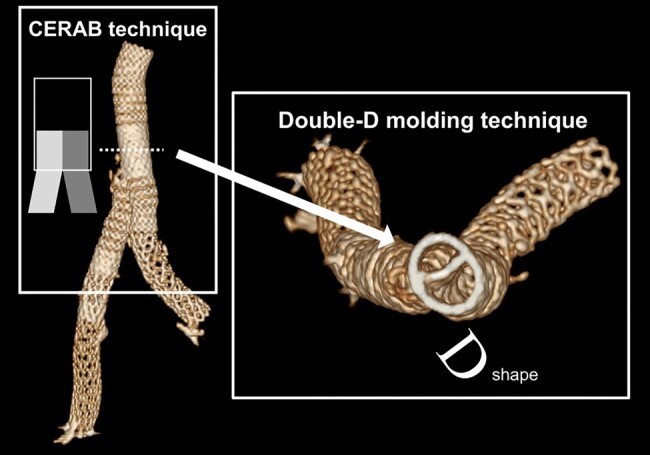


## Case presentation

A 74-year-old diagnosed with Stanford type B aortic dissection 15 years ago in other country presented with chronic bilateral lower extremity claudication. He had come back to home country and been referred to our hospital. The patient had history of hypertension, dyslipidaemia, chronic renal disease, and cerebral artery aneurysm. He was treated by medication and followed up by yearly CT angiography.

**Figure 1:**
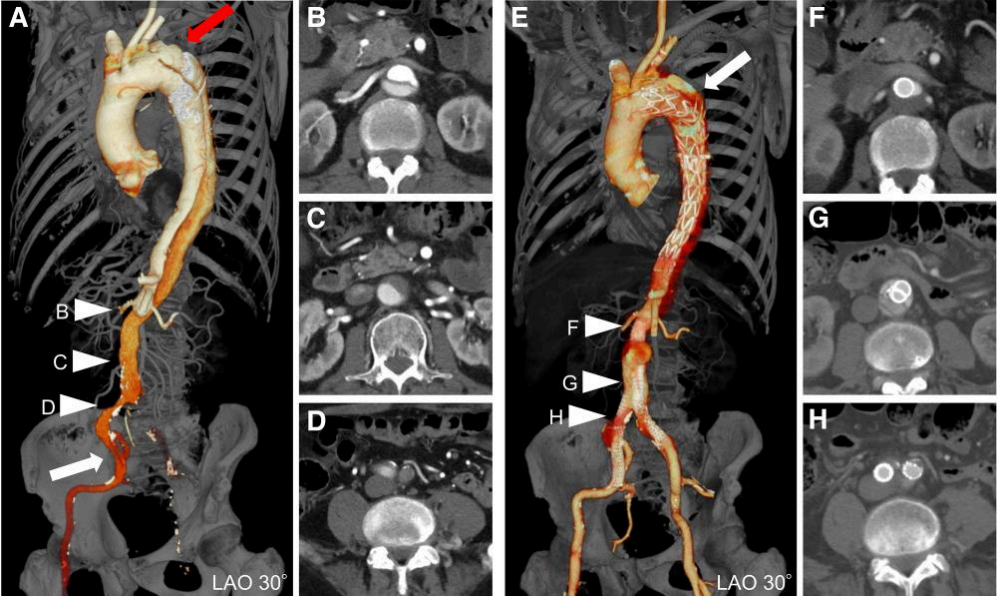
Computed tomography of pre- and post-hybrid endovascular therapy. (*A*) The large proximal entry was distal to the left subclavian artery (arrow). The true lumen of infrarenal aorta and bilateral common iliac arteries was totally collapsed by false lumen (arrowheads). The distal re-entry was distal to the right internal iliac artery (arrow). (*E*) The thoracic stent grafts were deployed (arrow) by transfemoral approach. The true lumen reconstruction by utilizing CERAB and DDMT was performed at abdominal aortic bifurcation (arrowheads).

CT angiography revealed the complex anatomy of aortic dissection (*Figure 1A*). The large entry tear was located at distal to the left subclavian artery, and dissection extended through visceral segment into bilateral iliac arteries. True lumen of infrarenal aorta and bilateral common iliac arteries was totally collapsed by false lumen, and the single re-entry tear was open at the right external iliac artery. A robust collateral connection into left lower extremity was established involving visceral and lumber arteries. The thoracic aneurysmal degeneration has progressed up to 6.2 cm diameter and eventually required repair. The value of ankle-brachial index in right and left limb was 0.88 and 0.76, respectively. Aortic catheter angiography was performed to elucidate aortoiliac blood flow. His right lower extremity was perfused by false lumen through the external iliac re-entry. His left lower extremity was perfused by collateral vessels via lumbar arteries to intrapelvic arteries. Bilateral leg flow was seriously insufficient.

**Figure 2:**
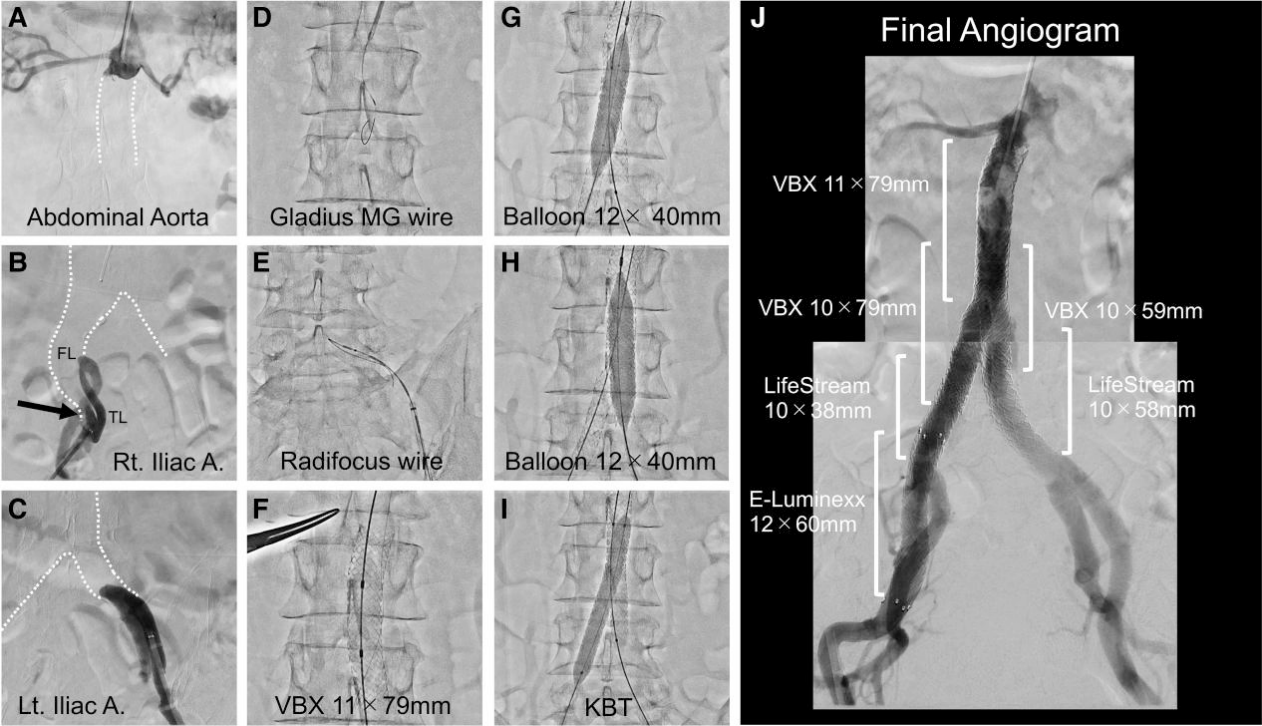
Endovascular recanalization for aortoiliac true lumen obstruction. (*A–C*) The pre-procedural angiogram. FL, false lumen; TL, true lumen. (*D*, *E*) The recanalization of true lumen by using looped wire technique. (*F–I*) The CERAB and DDMT procedures. KBT, kissing balloon technique. (*J*) The final angiogram demonstrated minimal type Ⅲ endoleak.

The patient was planned for two-stage endovascular therapy. Initially, we performed recanalization to the collapsed true lumen (*Figure 2*). Bidirectional approach was taken from right brachial and bifemoral arteries. We recanalized through intraluminal tracking by utilizing bidirectional looped wire technique with 0.014 inch Gladius MG14 (Asahi Intec, Aichi, Japan) and 0.035 inch Radifocus Guide Wire (Terumo, Tokyo, Japan). After guide wires traversed the obliterated segment, we confirmed by intravascular ultrasound imaging catheter that both guide wires passed entirely through the true lumen. An 11 mm × 79 mm Viabhan VBX covered stent (WL Gore & Associates, Flagstaff, AZ, USA) was place at infrarenal aorta as a main tube followed by post-dilatation with 16 mm balloon. Subsequently, kissing stent technique with two 10 mm Viabhan VBX devices was performed in an overlapping fashion. The overlapping zone has clearance between main tube and two kissing covered stents. Each kissing stents were overexpanded by 12 mm × 40 mm balloon one by one to screen out the clearance. Kissing balloon was performed with two 8.0 mm size balloons, creating double D-shape covered stents. The artificial covered stent bifurcation was established by utilizing the covered endovascular reconstruction of aortic bifurcation (CERAB) technique and double D-shape moulding technique (DDMT). The combination of those techniques was definitely effective to minimize type Ⅲ endoleak from stent graft overlap. LifeStream stent grafts (BD Peripheral Intervention, Tempe, AZ, USA) were extended to internal iliac bifurcations. The false lumen of common iliac arteries was totally closed by stent graft implantation. The bare nitinol stent was added to exclude re-entry flap at right external iliac artery. We created a sufficient inner area within infrarenal covered stents to facilitate the deliverability of TEVAR system.

As secondary treatment, 1-debranching TEVAR with axillary artery bypass was successfully performed. The 24 Fr Gore DrySeal sheath was smoothly cannulated by right femoral approach via abdominal covered stents. The 38 to 34 mm × 50 and 28 to 24 mm × 150 mm Medtronic Valiant Captivia stent grafts (Medtronic, Santa Rosa, CA, USA) were deployed in descending aorta through the Gore DrySeal sheath. The thoracic stent graft was proximally extended with Zenith Alpha Extension (Cook Medical, Bloomington, IN, USA) to seal the ostium of left subclavian artery (*Figure 1E*).

**Figure 3:**
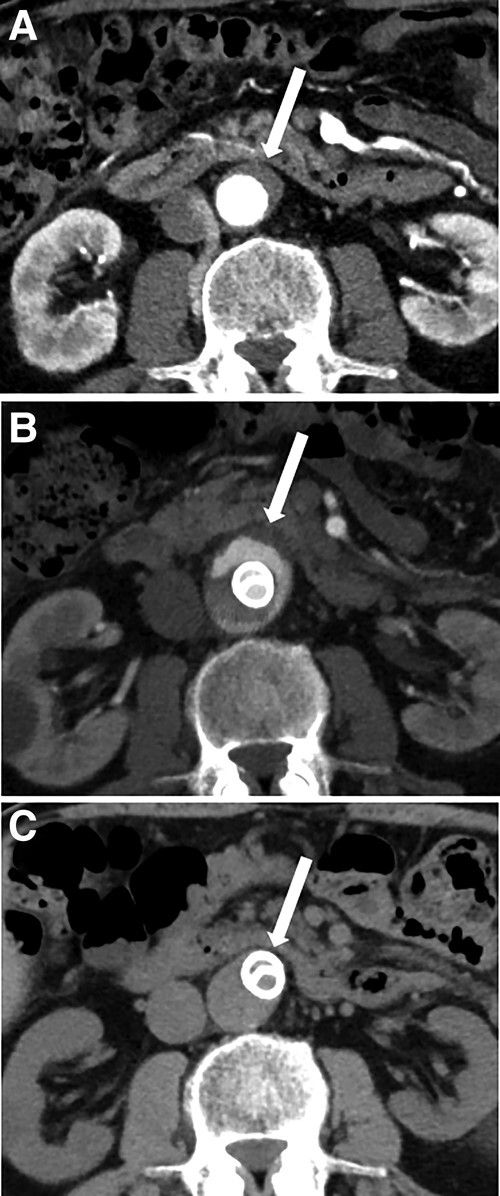
Abdominal aneurysmal regression in follow-up CT images. (*A*) The true lumen of infrarenal aorta was totally collapsed before the procedures (arrow). (*B*) CT angiography demonstrated aneurysmal formation and type Ⅲ endoleak in the true lumen after the TEVAR procedure. (*C*) True lumen aneurysm regressed at 1-year follow-up.

The patient’s symptom of lower extremities was fully resolved and the value of ankle-brachial index changed into normal level, 1.04 and 1.03 for the right and left sides, respectively. A minor type Ⅲ endoleak manifested in the angiographic assessment following infrarenal reconstruction (*Figure 2J*). Nevertheless, successive CT images elucidated regression of abdominal aneurysm (*Figure 3*). Although we carefully considered intervention for type Ⅲ endoleak in the course of follow-up, CT images demonstrated the complete resolution of endoleak. Moreover, no stent graft-induced new entry tear was observed following infrarenal reconstruction.

## Discussion

The case of chronic aortic dissection with infrarenal true lumen obliteration is quite rare. Nevertheless of lower extremity symptoms, that patient did not treated by surgical or endovascular treatment. Although the thoracic aortic aneurysmal degeneration progressed, the aortic true lumen obstruction disabled TEVAR option due to the loss of transfemoral approach route. This is the first case report that CERAB and DDMT followed by transfemoral TEVAR were performed for chronic aortic dissection with true lumen obliteration. This combined with CERAB and DDMT was absolutely effective to minimize type Ⅲ endoleak in infrarenal segment. Thoracic endovascular aortic repair was safely performed via femoral approach through revascularized true lumen. The two-stage endovascular strategy offered minimally invasive therapy to the patient.

In a patient with thoracic aneurysmal degeneration concomitant with infrarenal true lumen obliteration, the traditional approach is open surgical thoracic repair and extra-anatomic bypass grafting. However, the graft patency of extra-anatomic bypass such as axillofemoral bypass is not reliable. Graft failure results in serious sudden ischaemia of bilateral lower extremities. Since the 1990s, less invasive treatments employing an endovascular approach have been proposed. Occlusion of proximal entry tear with stentgraft and endovascular fenestration are the main strategy.^4^ In this case, TEVAR with endovascular fenestration does not offer sufficient perfusion to left lower extremity. Therefore, we certainly expected that anatomical recanalization was the most reasonable treatment strategy.

The CERAB technique was developed in an attempt to overcome the radial mismatch of kissing stents when treating aortoiliac occlusive disease.^5^  *In vitro* study showed that CERAB configuration had a geometric advantage when compared with kissing stents configuration.^6^ However, mal-apposed covered stents in overlapping site might result in type Ⅲ endoleak in the case with aneurysm. The DDMT was also developed to establish less radial mismatch of kissing covered stents.^7^ Moreover, the DDMT could prevent type Ⅲ endoleak by filling the gaps within overlapping covered stents. Since this case had aortic dissection aneurysm, massive endoleak will lead to progressive aneurysmal dilatation. Consequently, the combination of two techniques minimizes mal-apposition of overlapping covered stents and type Ⅲ endoleak and abdominal true lumen dilatation regressed at follow-up point. The transfemoral approach via abdominal CERAB configuration was safe and feasible during TEVAR procedure. Completion of thoracic and abdominal aortic reconstruction developed normal perfusion into lower extremities and remodelled aortic dissection aneurysm.

## Conclusion

We successfully treated thoracic aneurysmal degeneration concomitant with abdominal aortic true lumen obliteration by two-stage endovascular therapy, i.e. CERAB and DDMT followed by transfemoral TEVAR.

## Data Availability

The data underlying this article are available in the article.

## Lead author biography

Dr Kuniyasu Ikeoka serves as an interventional specialist at NHO Osaka National Hospital, Osaka, Japan. He obtained his medical degree from Medical School of Osaka University, Osaka, Japan. Subsequently, he pursued his post-graduate training at Kansai Rosai Hospital, Hyogo, Japan. He completed a doctoral programme in medical science at Osaka University. His main interests are innovative transcatheter vascular therapy and advanced vascular imaging techniques.

**Consent**: The authors confirm that written consent for submission and publication of this case report including images and associated text has been obtained from the patient in line with COPE guidance.

**Funding:** None declared.
